# The Influence of Regiochemistry on the Performance of Organic Mixed Ionic and Electronic Conductors

**DOI:** 10.1002/anie.202304390

**Published:** 2023-06-12

**Authors:** Roman Halaksa, Ji Hwan Kim, Karl J. Thorley, Peter A. Gilhooly‐Finn, Hyungju Ahn, Achilleas Savva, Myung‐Han Yoon, Christian B. Nielsen

**Affiliations:** ^1^ Department of Chemistry Queen Mary University of London Mile End Road London E1 4NS UK; ^2^ School of Materials Science and Engineering Gwangju Institute of Science and Technology (GIST) 123 Cheomdangwagi-ro, Buk-gu Gwangju 61005 Republic of Korea; ^3^ Center for Applied Energy Research University of Kentucky Lexington KY 40511 USA; ^4^ Pohang Accelerator Laboratory, POSTECH Pohang 37673 Republic of Korea; ^5^ Department of Chemical Engineering and Biotechnology University of Cambridge Cambridge CB3 0AS UK

**Keywords:** Mixed Ionic-Electronic Conductors, Organic Bioelectronics, Organic Electrochemical Transistors, Regiochemistry, Semiconducting Polymers

## Abstract

Thiophenes functionalised in the 3‐position are ubiquitous building blocks for the design and synthesis of organic semiconductors. Their non‐centrosymmetric nature has long been used as a powerful synthetic design tool exemplified by the vastly different properties of regiorandom and regioregular poly(3‐hexylthiophene) owing to the repulsive head‐to‐head interactions between neighbouring side chains in the regiorandom polymer. The renewed interest in highly electron‐rich 3‐alkoxythiophene based polymers for bioelectronic applications opens up new considerations around the regiochemistry of these systems as both the head‐to‐tail and head‐to‐head couplings adopt near‐planar conformations due to attractive intramolecular S−O interactions. To understand how this increased flexibility in the molecular design can be used advantageously, we explore in detail the geometrical and electronic effects that influence the optical, electrochemical, structural, and electrical properties of a series of six polythiophene derivatives with varying regiochemistry and comonomer composition. We show how the interplay between conformational disorder, backbone coplanarity and polaron distribution affects the mixed ionic‐electronic conduction. Ultimately, we use these findings to identify a new conformationally restricted polythiophene derivative for p‐type accumulation‐mode organic electrochemical transistor applications with performance on par with state‐of‐the‐art mixed conductors evidenced by a μC* product of 267 F V^−1^ cm^−1^ s^−1^.

## Graphical abstract

Investigating the influence of sidechain regiochemistry on a series of thiophene‐based mixed ionic‐electronic conducting polymers, the degree of conformational disorder is found to be a crucial indicator of performance. Maximising non‐covalent planarising interactions and polaronic charge delocalisation during molecular design must consequently be carefully weighed against considerations around conformational restrictions.

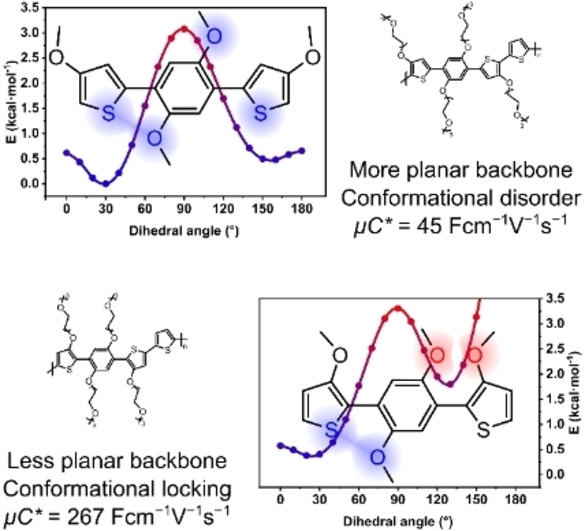

## Introduction

Semiconducting materials have long played a key role in modern society. Organic electronics have recently seen rapid developments, especially for applications such as organic light emitting diodes,[^1^, ^2^] organic photovoltaic cells,[^3^, ^4^] and organic transistors.[^5^, ^6^] Thanks to the significantly higher biocompatibility of organic molecules compared to inorganic silicon,^[7]^ in the field of bioelectronics major advancements have occurred as well. For instance, ion, and metabolite sensing,[^8^, ^9^] cells influencing and drug delivery using ion pumps,[^10^, ^11^] and fabrication of neuromorphic devices[^12^, ^13^] have been successfully achieved.

The organic electrochemical transistor (OECT) is very similar in construction to the organic field‐effect transistor with two fundamental differences. In the case of the OECT, the gate electrode is interfaced to the active layer via an electrolyte droplet while the active layer allows for ion infiltration to compensate polaronic charges and enable bulk doping/dedoping. Since mentioned electrolyte can also be aqueous, an OECT represents an ideal platform for interfacing organic electronics with biological and artificial systems.[^14^, ^15^] If a bias is applied to the gate electrode, ions from the electrolyte are injected into the active layer. Due to the necessity of charge balance, a hole from the source electrode or an electron from the drain electrode is simultaneously fed into the conjugated backbone of the active layer material. This process makes the active layer conductive in the case of accumulation mode (non‐conductive in the case of depletion mode).^[16]^ It follows from the principle of electrochemical doping that materials suitable for OECT applications must have a combination of ionic and electrical conductivity capabilities, and when preparing new bioelectronic materials, adequate attention must be paid to both properties.

The most widely used material to produce OECTs is the intrinsically conducting blend of poly(3,4‐ethylenedioxythiophene) and polystyrene sulfonate (PEDOT : PSS). This material also has the current primacy in the Figure of merit of OECT performance (μC*=1500 F cm^−1^ V^−1^ s^−1^).^[17]^ However, an OECT fabricated with PEDOT : PSS works in depletion mode which at low gate bias causes an inferior on/off ratio.^[18]^ Another problem is its acidic nature, which limits the stability of the devices.^[19]^ Since chemical modifications of PEDOT : PSS are relatively challenging, it is difficult to easily remedy the disadvantages mentioned. Due to these detriments, significant progress has been made in recent years on developing p‐type (hole transport) accumulation mode mixed ionic‐electronic conductors with especially polythiophene‐based materials showing excellent performances (μC*>500 F V^−1^ cm^−1^ s^−1^),[^20^, ^21^] but also donor‐acceptor systems emerging as viable candidates.[^22^, ^23^, ^24^]

**Figure 1:**
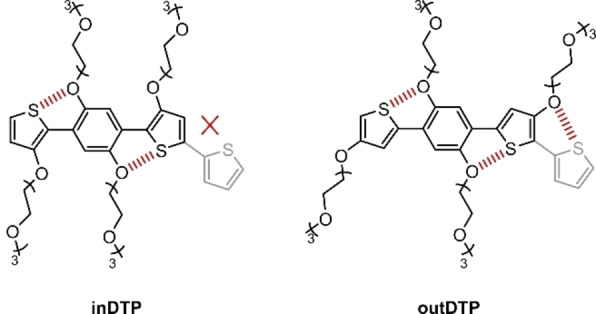
Chemical structures of the two DTP regioisomers depicted with a thiophene comonomer (light grey) and attractive intramolecular S−O interactions highlighted (red dashed lines).

Here in this work, we have chosen the electron‐rich 1,4‐dithienylphenylene (DTP) moiety bearing four triethylene glycol side chains shown in Figure 1 as a platform to investigate the effect of side chain regiochemistry, electron density, and backbone planarity in p‐type polymers for OECT applications.^[25]^ The hydrophilic oligoether side chains ensure the necessary interaction of the polymers with the solvated ions during OECT operation,^[26]^ and, owing to their attachment to the aromatic cores directly by the oxygen atom, they also increase the electron density of the DTP building block. High electron density is a prerequisite for enabling oxidation (and conversion to a conductive state) at a low gate bias. Both DTP structures shown in Figure 1 can form two attractive non‐covalent interactions between the oxygen atoms on the central phenylene unit and the sulfur atoms of the flanking thiophene units.[^27^, ^28^] These interactions are likely to support the planarisation of the structure, which could enhance intermolecular π‐stacking interactions and thus the macroscopic charge transport properties.

The effect of glycol chain length on the performance of mixed ionic‐electronic conductors has been investigated several times. Attention was paid to the length of the glycol side chains itself^[29]^ and to materials that combine building blocks with different side chain lengths in their structure.[^20^, ^30^] On the other hand, the effect of the regiochemistry of the glycol side chain on the conjugated backbone has rarely been studied.^[21]^ Therefore, two different regioisomers of the DTP building block were prepared as depicted in Figure 1. The difference is the position of the glycol side chains on the flanking thiophene cores. In the inDTP version, these glycol side chains face towards the central phenylene core, whereas in the outDTP version, they face away from it. While both isomers can form two planarising S−O interactions involving the phenylene unit, the outDTP isomer can also form planarising interactions with a sulfur‐containing co‐monomer (for example thiophene as indicated in Figure 1) whereas the inDTP isomer importantly cannot.

**Figure 2:**
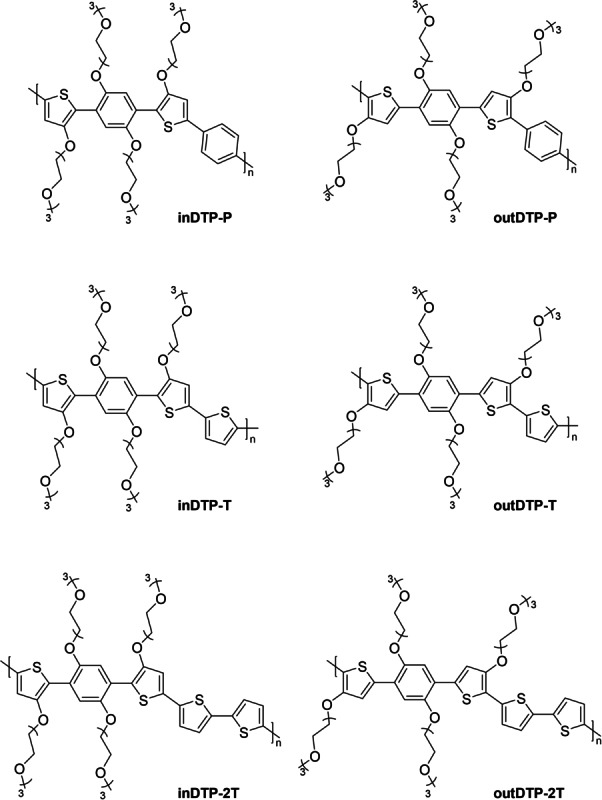
Chemical structures of the target polymers.

As such, these two regioisomers and their various copolymers offer an opportunity to investigate how regiochemistry and associated conformational restrictions caused by attractive non‐covalent interactions affect macroscopic properties related to mixed ionic‐electronic conduction including structural order and charge transport. Moreover, the electronic contribution from the electron‐donating side chains will differ for the two regioisomers which is likely to affect the polaron delocalisation found to play an important role in OECT charge transport.[^21^, ^31^] To perform this investigation thoroughly across a larger series of copolymers, we have copolymerised the two isomeric DTP building blocks with 1,4‐phenylene (P), thiophene (T) and 2,2′‐bithiophene (2T) to afford the six polymers depicted in Figure 2. The choice of comonomer provides a variation in electron‐rich character (T and 2T more electron‐rich than P), in glycol side chain density per repeat unit length (longer 2T unit reduces side chain density), and in the availability of additional S−O interactions for the outDTP system (none in the case of outDTP‐P).

## Results and Discussion

### Polymer synthesis

**Scheme 1:**
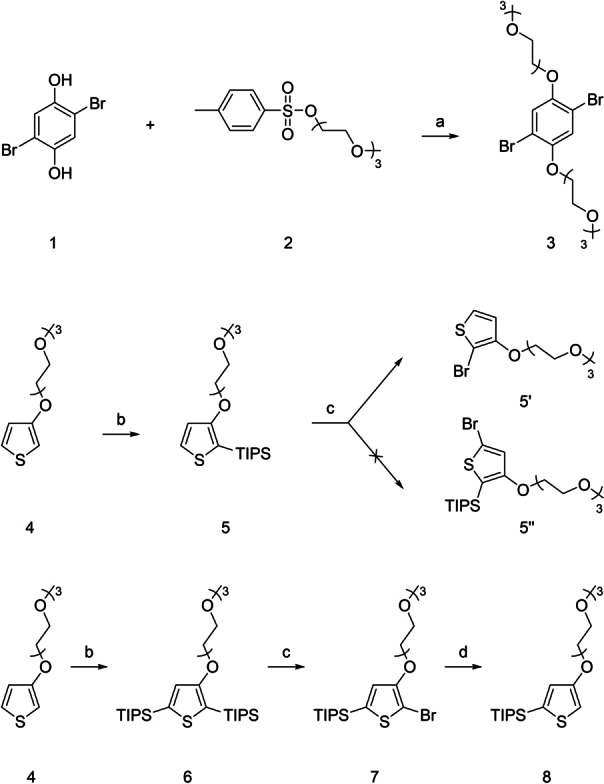
Synthetic strategy for the synthesis of the basic phenylene and thiophene building blocks 3, 5, and 8. a) K_2_CO_3_, 70 °C, DMF; b) 1. BuLi, −78 °C, 2. TIPSCl, RT, THF; c) NBS, 0 °C, DMF; d) 1. BuLi, −78 °C, 2. MeOH, RT, THF.

As illustrated in Scheme 1, the synthesis of the central phenylene moiety (compound **3**) was performed by reacting compounds **1** and **2** in anhydrous *N*,*N*‐dimethylformamide in an inert atmosphere at 70 °C in the presence of potassium carbonate overnight. Unlike most other papers mentioning the synthesis of this compound, initial purification of the crude product was performed by filtration through a silica pad (hexane/ethyl‐acetate 1/1). The obtained product was then dissolved in a mixture of ethanol and water and heated at 70 °C overnight in the presence of potassium hydroxide. The pure product was subsequently obtained by simple extraction (diethyl ether/water) in a yield of 46 %. The synthesis of the thiophene building block (**5**), protected in the 2‐position, was performed using a literature procedure in a yield of 89 %.^[32]^ The synthesis of the isomeric building block (**8**), protected in the 5‐position, was made possible by the anomalous reactivity of compound **5**. This, by reaction with *N*‐bromosuccinimide, does not provide the expected product brominated in the 5‐position (**5′′**), but the replacement of the silyl protecting group in the 2‐position with a bromine atom takes place instead, which forms compound **5′**. Therefore, molecule **6** was prepared in a yield of 72 % by double lithiation of molecule **4** with *n*‐butyllithium in anhydrous tetrahydrofuran under nitrogen at −78 °C, followed by quenching of the reaction mixture with triisopropylsilyl (TIPS) chloride. The obtained molecule **6** was subsequently reacted with *N*‐bromosuccinimide in anhydrous and degassed *N*,*N*‐dimethylformamide at 0 °C overnight in a light‐protected apparatus. Following the example of the previously mentioned anomaly, this led to the TIPS protecting group in the 2‐position being replaced by a bromine atom affording compound **7**. The product was isolated in 90 % yield. However, if the solvent was not degassed, the reaction proceeded non‐selectively and the TIPS group was replaced not only in the 2‐position but also in the 5‐position. Compound **7** was subsequently lithiated in anhydrous tetrahydrofuran under nitrogen at −78 °C. The reaction mixture was quenched with methanol, and compound **8** (positional isomer of compound **5**) was obtained in a yield of 81 %.

**Scheme 2:**
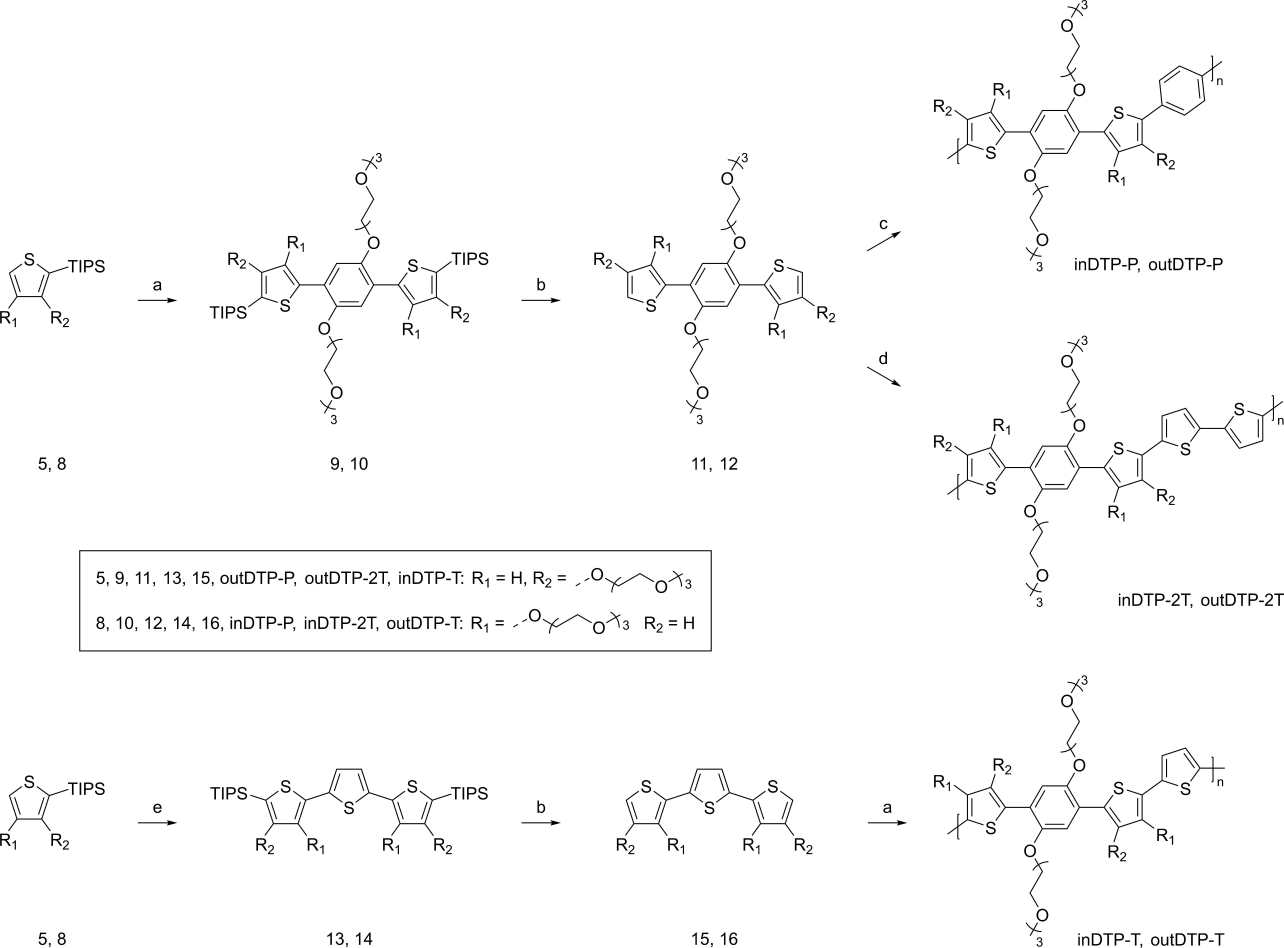
Synthetic strategy for the synthesis of the target polymers inDTP‐P, outDTP‐P, inDTP‐2T, outDTP‐2T, inDTP‐T, and outDTP‐T. Direct Arylation (DA): Pd(OAc)_2_, tri(2‐methoxyphenyl)phosphine, Cs_2_CO_3_, caesium pivalate, 120 °C; a) compound 3, DA, toluene; b) TBAF, RT, THF; c) 1,4‐dibromobenzene, DA, toluene; d) 5,5′‐dibromo‐2,2′‐bithiophene, DA, chlorobenzene; e) 2,5‐dibromothiophene, DA, toluene.

As outlined in Scheme 2, a direct arylation reaction with compound **3** was performed in anhydrous and degassed toluene at 120 °C for 80 h using either thiophene derivative **5** or **8**. The reaction was catalysed by a mixture of palladium acetate, tris (*o*‐methoxyphenyl)phosphine, caesium carbonate and caesium pivalate. This procedure originally used pivalic acid as the carboxylic ligand.^[32]^ However, on contact with caesium carbonate, it was releasing water, which caused the coagulation of salts necessary for catalysis. Replacement of pivalic acid with caesium pivalate improved the reaction yields by about 10 %. The two DTP moieties **9** (outDTP) and **10** (inDTP) were prepared by the above reaction in 86 % and 73 % yield, respectively. Deprotection with tetrabutylammonium fluoride at room temperature in anhydrous tetrahydrofuran afforded molecules **11** (89 % yield) and **12** (71 % yield). In the last synthetic step, compounds **11** and **12** were polymerised by a direct arylation reaction with 1,4‐dibromobenzene using the same catalytic system and with 5,5′‐dibromo‐2,2′‐bithiophene (same catalytic system was employed except with chlorobenzene as solvent). Four polymers: inDTP‐P (dark red solid, 84 %, *M*_n_ 46 kg mol^−1^), outDTP‐P (dark red solid, 80 %, *M*_n_ 22 kg mol^−1^), inDTP‐2T (black solid, 68 %, *M*_n_ 13 kg mol^−1^) and outDTP‐2T (79 %, *M*_n_ 16 kg mol^−1^) were obtained. While molecules **11** and **12** reacted readily with 1,4‐dibromobenzene and 5,5′‐dibromo‐2,2′‐bithiophene to afford the DTP‐P and DTP‐2T polymers, the corresponding reactions with 2,5‐dibromothiophene proceeded in very low yields preventing DTP‐T synthesis following this route. This explains the alternative strategy devised (Scheme 2), generating first molecules **13** and **14** in low yields (16 % and 37 %, respectively) by reaction with 2,5‐dibromothiophene, after which deprotection and reaction with molecule **3** afforded the two DTP‐T polymers: inDTP‐T (dark blue solid 72 %, *M*_n_ 28 kg mol^−1^) and outDTP‐T (dark blue solid 19 %, *M*_n_ 19 kg mol^−1^). Purification of all obtained polymers was performed by precipitation in methanol, filtration into the Soxhlet thimble and washing with hexane, methanol, and acetone. Subsequently, the polymers were isolated from the chloroform‐soluble fraction. Synthetic details and full molecular weight characterisation (Table S1) can be found in the Supporting Information. Although significantly different degrees of polymerisation are obtained across the full polymer series, we note that less variation occurs when comparing regioisomer pairs with identical comonomer unit (e.g., inDTP‐T vs. outDTP‐T). Good thermal stability of the polymers was confirmed via thermogravimetric analysis which showed 5 % mass loss above 330 °C for all six polymers (Supporting Information Section 5). Differential scanning calorimetry revealed endothermic thermal transitions for the two DTP‐P polymers (≈205 °C for inDTP‐P and ≈175 °C for outDTP‐P) and for inDTP‐T (≈165 °C), whereas the three other polymers showed no discernible thermal events (Supporting Information Section 6).

### Optical properties

**Figure 3:**
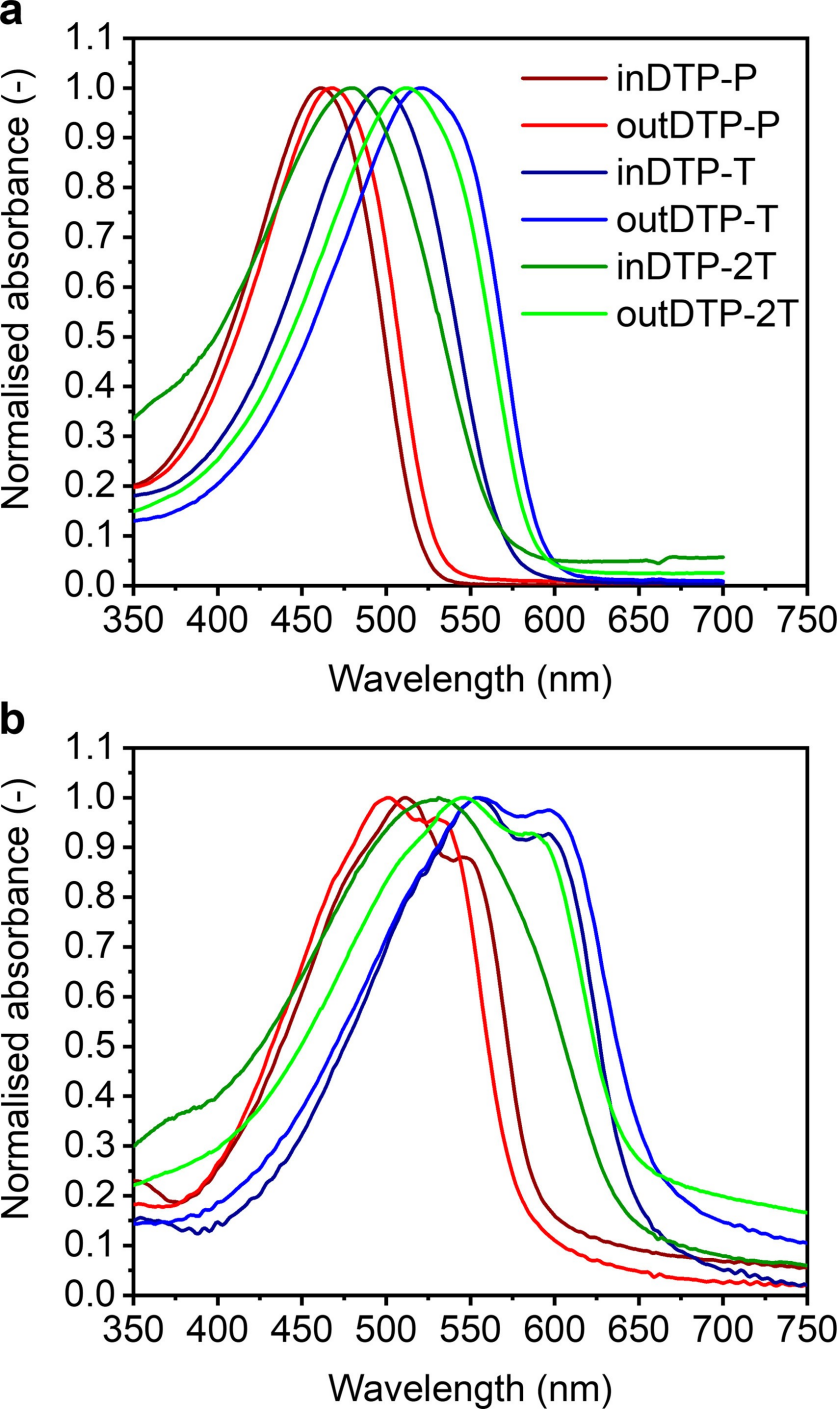
Normalized UV/Vis spectra of the six polymers [a] chloroform solution (3 μg mL^−1^), [b] thin film on glass slide spin‐cast from 5 mg mL^−1^ chloroform solution.

Characterisation of the prepared polymers began by measuring UV/Vis absorption spectra in chloroform solution (Figure 3a, Table 1). All polymers showed absorption in the visible part of the electromagnetic spectrum, with absorption maxima in the range 461–521 nm. The most red‐shifted absorption was observed for the outDTP‐T polymer (*λ*_max_ 521 nm), followed by the outDTP‐2T polymer (*λ*_max_ 512 nm). Slightly blue‐shifted absorption profiles were observed in case of inDTP‐T (*λ*_max_ 496 nm) and inDTP‐2T (*λ*_max_ 481 nm). The electron‐poorer outDTP‐P polymer was further blue‐shifted with *λ*_max_ 468 nm, followed by the inDTP‐P polymer with *λ*_max_ 461 nm. The “in” polymers appear to be less ordered with a lower effective conjugation length in solution than the corresponding “out” polymers; this could be ascribed to fewer planarising S−O contacts (Figure 1) as further investigated computationally (vide infra).

**Table 1:** Optical properties of the target polymers in chloroform solution and thin film.

Polymer	*λ*_max_ solution [nm]	*λ*_max_ film [nm]	Absorption onset [nm]	Optical band gap [eV]
inDTP‐P	461	512	596	2.08
outDTP‐P	468	503	579	2.14
inDTP‐T	496	555	656	1.89
outDTP‐T	521	555	670	1.85
inDTP‐2T	481	531	653	1.90
outDTP‐2T	512	545	658	1.88

UV/Vis spectroscopy performed on thin films deposited on glass slides by spin‐coating showed an altered trend (Figure 3b, Table 1). The most red‐shifted polymers here are the two thiophene copolymers inDTP‐T and outDTP‐T both with *λ*_max_ 555 nm and with shoulders at 597 nm. Next there was outDTP‐2T polymer with *λ*_max_ 545 nm with a shoulder at 582 nm, followed by inDTP‐2T with *λ*_max_ 531 nm and no observable vibronic fine structure. The two phenylene copolymers, however, changed order here compared to the solution spectra with inDTP‐P (*λ*_max_ 512 nm with a shoulder 548 nm) red‐shifted relative to outDTP‐P (*λ*_max_ 503 nm with a shoulder 530 nm). Going from solution to the solid state, all the “in” polymers showed larger red‐shifts (50–59 nm) than the “out” polymers (33–35 nm) indicating that the larger degree of disorder in solution for the “in” polymers does not prevent a good degree of ordering in the solid state. The significant red‐shift of the DTP‐T polymers and slight red‐shift of the DTP‐2T polymers relative to the DTP‐P polymers, which is visible in both solution and thin film measurements, can be explained by the fact that the DTP‐T and DTP‐2T polymers are more planar than the DTP‐P polymers, arguably both due to less steric hindrance and greater quinoidal character with the less aromatic and smaller thiophene unit(s).The optical band gaps extracted from thin film absorption onsets fall in the range of 1.85–2.14 eV with the following trend outDTP‐T 1.85 eV, outDTP‐2T 1.88 eV, inDTP‐T 1.89 eV, inDTP‐2T 1.89 eV, inDTP‐P 2.08 eV and outDTP‐P 2.14 eV.

### Electrochemical properties

**Figure 4:**
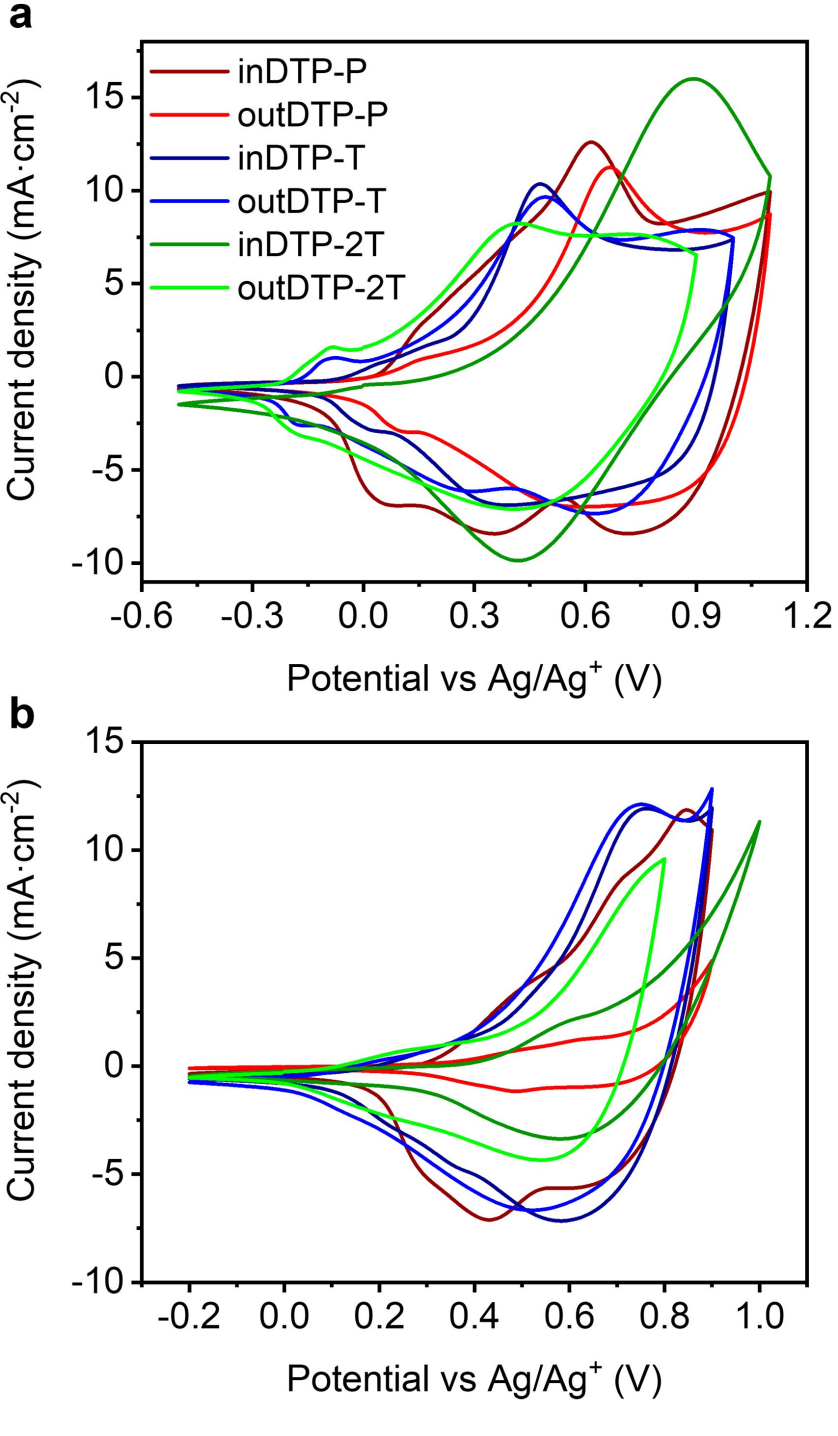
Cyclic voltammograms of thin polymer films on glassy carbon electrode drop‐cast from chloroform solution (5 mg mL^−1^) with a scan rate of 50 mV s^−1^ versus Ag/Ag^+^. [a] with 0.1 M TBAPF_6_ as supporting electrolyte in acetonitrile, [b] with 0.1 M NaCl as supporting electrolyte in water.

Electrochemical properties were studied using cyclic voltammetry (CV) in a thin film arrangement. The polymer layer was drop‐cast on the glassy carbon electrode (working electrode) from a 5 mg mL^−1^ chloroform solution. A platinum wire served as the counter electrode and Ag/AgNO_3_ (acetonitrile) as the reference electrode; tetrabutylammonium hexafluorophosphate (TBAPF_6_) (0.1 M solution in acetonitrile) was used as the supporting electrolyte. All polymers showed clear anodic and cathodic peaks under the given conditions as depicted in Figure 4a. The onset of oxidation of all polymers was obtained from the CV measurements and used to estimate the ionisation potential (IP) and subsequently the electron affinity (EA) by subtracting the optical band gaps (Table 2).

**Table 2:** Electrochemical properties from cyclic voltammetry performed in 0.1 M TBAPF_6_ acetonitrile solution at a scan rate of 50 mV s^−1^.

Polymer	*E*_onset_ [V]	IP [eV]	EA [eV]
inDTP‐P	0.04	4.46	2.38
outDTP‐P	0.02	4.44	2.29
inDTP‐T	−0.08	4.34	2.45
outDTP‐T	−0.18	4.24	2.39
inDTP‐2T	−0.12	4.30	2.40
outDTP‐2T	−0.22	4.20	2.32

The IP values were practically identical for inDTP‐P and outDTP‐P with values of 4.46 eV and 4.44 eV, respectively. The DTP‐T and DTP‐2T polymers with the more electron‐rich thiophene units showed slightly lower IPs in the range 4.20–4.34 eV. The already mentioned ability of the outDTP‐T and outDTP‐2T polymers to form additional attractive S−O interactions between the glycolated thiophene core and the unsubstituted thiophene co‐monomer making the whole system more planar is manifested in slightly lower IP values for the “out” polymers compared to the corresponding “in” polymers. Such planarising interactions are not present for the two phenylene copolymers, explaining the very similar IP values of inDTP‐P and outDTP‐P. Experimental IP values are generally in good agreement with values obtained from computational simulations (Table 5).

To better assess the behaviour of the materials in an OECT relevant environment, the thin film CV measurement was also performed in a 0.1 M aqueous solution of NaCl with Ag/AgCl (water) reference electrode as illustrated in Figure 4b. In the range of −0.2–0.9 V (inDTP‐P, outDTP‐P, inDTP‐T and outDTP‐T), −0.2–0.8 V (outDTP‐2T), and −0.2–1.0 V (inDTP‐2T), all materials showed anodic currents indicative of oxidation with excellent electrochemical stability evidenced by minimal degradation during repeated cycling over 100 scans (Supporting Information Section 7). In aqueous electrolyte, the onset of oxidation was generally observed at higher potentials (Table 3) with the phenylene copolymers again showing comparable values of 0.31 V (inDTP‐P) and 0.34 V (outDTP‐P). The T and 2T copolymers showed onsets of oxidation at 0.14 V (inDTP‐T), 0.08 V (outDTP‐T), 0.43 V (inDTP‐2T), and 0.09 V (outDTP‐2T) with the “out” polymers again showing earlier onsets as discussed above.

**Table 3:** Onsets of oxidation extracted from cyclic voltammetry (CV; Figure 4b) and spectroelectrochemistry (SP; Figure 5 and Supporting Information Section 8) data in 0.1 M aqueous NaCl solution.

Polymer	*E*_onset, cv_ [V]	*E*_onset, SP_ [V]
inDTP‐P	0.31	0.32
outDTP‐P	0.34	0.42
inDTP‐T	0.14	0.22
outDTP‐T	0.08	0.10
inDTP‐2T	0.43	0.28
outDTP‐2T	0.09	0.12

**Figure 5:**
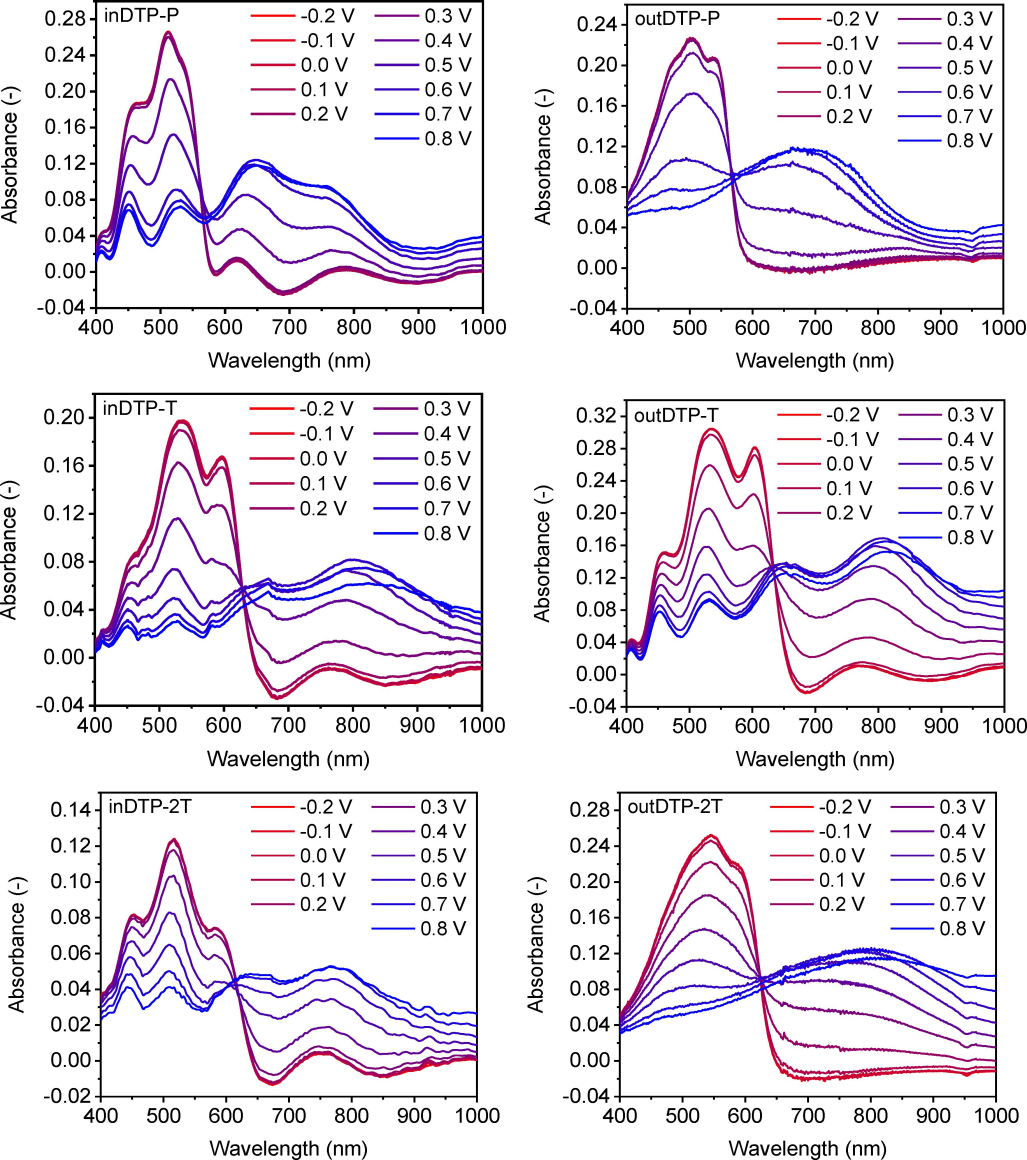
Spectroelectrochemistry of the target polymers spin‐cast from 5 mg mL^−1^ chloroform solution onto indium tin oxide coated glass slides in 0.1 M NaCl water solution.

Since all the polymers showed the ability to oxidise in aqueous electrolyte, they were subsequently subjected to spectroelectrochemistry measurements. The given method is used to investigate the oxidation of the bulk polymer thin film manifested by quenching of the π‐π* transition with the simultaneous formation of polaron (and bipolaron) absorption bands. The experiments were again performed in 0.1 M aqueous NaCl solution with a Ag/AgCl reference electrode. Polymer thin films were spin‐cast onto indium tin oxide coated glass slides from chloroform solution (5 mg mL^−1^) and immersed in the electrolyte in a spectroelectrochemical cell. Subsequently, the applied potential was raised from −0.2 V to 0.8 V in steps of 0.1 V. All polymers showed gradual quenching of the π‐π* transition during the stepwise oxidation with simultaneous appearance of a polaron peak (inDTP‐P 648 nm, outDTP‐P 693 nm, inDTP‐2T 766 nm, outDTP‐2T 801 nm, inDTP‐T 799 nm, outDTP‐T 805 nm) as well as indications of bipolaron formation at higher potentials as shown in Figure 5. Finally, the potential was returned to a value of −0.2 V in order to test the reversibility of the process; restoration of the π‐π* absorption band indicating reversibility was observed in all cases (Supporting Information Section 8).

The oxidation onsets from the spectroelectrochemical measurement (Table 3, Supporting Information Section 8) were obtained by plotting the π‐π* absorbance as a function of potential. These values from films spin‐cast onto ITO‐coated glass are generally in good agreement with the cyclic voltammetry data from films drop‐cast onto the glassy carbon electrode. Again, we note that for the DTP‐T and DTP‐2T polymers, the “out” regioisomers with more planarising interactions have earlier onsets of oxidation than the “in” regioisomers. The spectroelectrochemical data indicate that the bulk polymer films are electroactive in the electrochemical window of water and hence suitable candidates for p‐type OECT operation.

### Transistor characterisation

**Figure 6:**
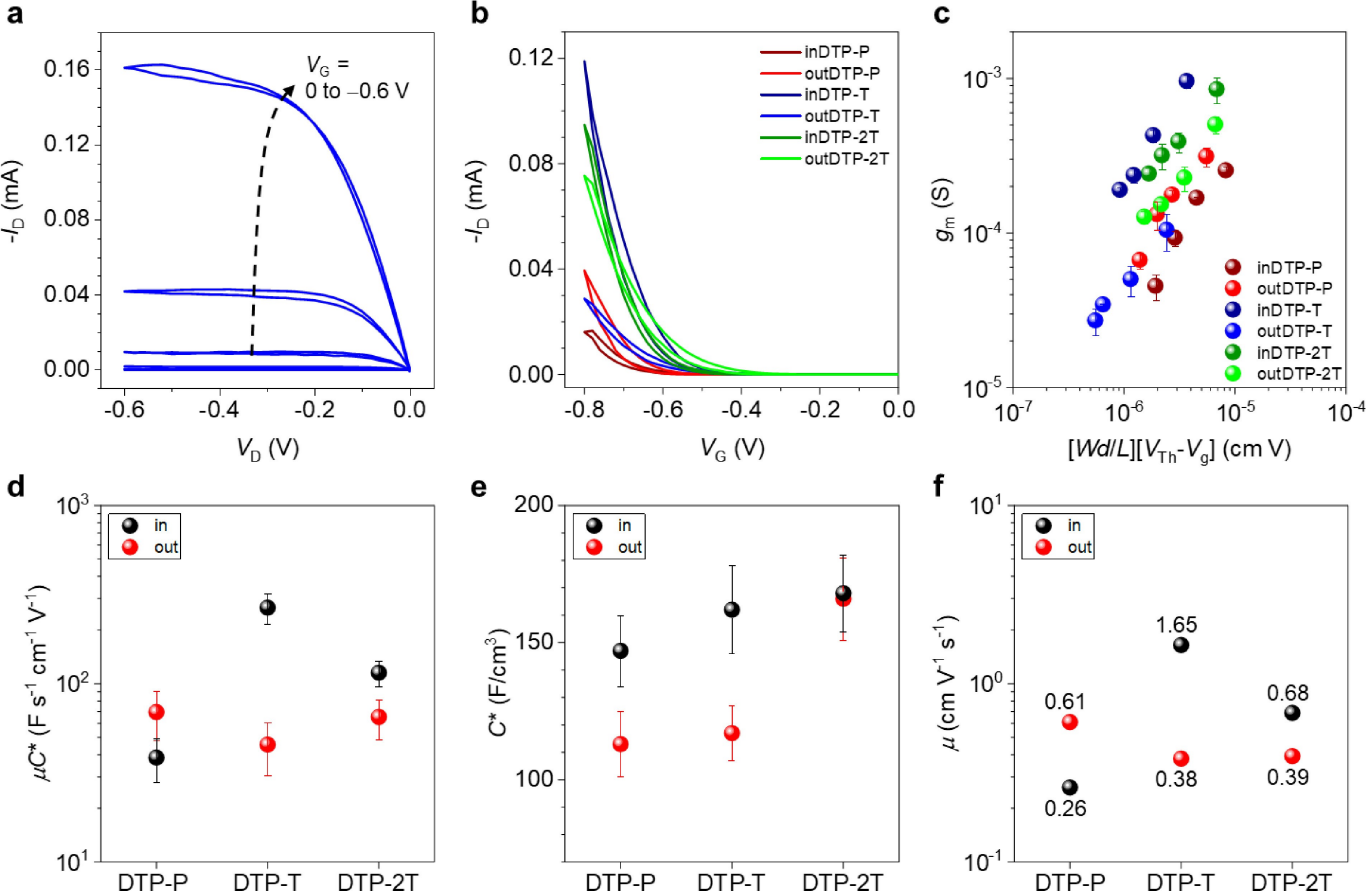
Electrical and electrochemical characterizations of DTP‐based polymer OECTs. a) Representative output curve of the inDTP‐T OECT. *V*_G_ was scanned from 0 to −0.8 V in the direction of the black dotted arrow. b) Transfer curves of DTP‐based polymer OECTs at *V*_D_=−0.6 V. c) Plot of transconductance as a function of channel dimensions and operational parameters. d) μC* value of each polymer extracted from the slope of plot (c). e) Plots of extracted C* of DTP‐based polymer films. f) Plots of the mobility values extracted by dividing μC* with C* obtained from EIS measurements.

Since all prepared materials showed excellent stability during measurement of cyclic voltammetry in aqueous electrolyte and subsequently the ability of reversible oxidation in spectroelectrochemistry, OECT characterisation followed, as the most important assessment of mixed ionic and electronic conducting properties. OECT devices with channel dimension of 80 μm×20 μm were fabricated by orthogonal patterning process using fluoropolymer, CYTOP™. The detailed fabrication procedure can be found in the Supporting Information (Supporting Information Section 9 and Figure S81). Device operation was conducted under N_2_ atmosphere, using a 0.1 M NaCl aqueous solution and a Ag/AgCl electrode as the gate electrode. OECT performance of devices incorporating the DTP polymers were interpreted using the following equation:
(1)

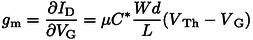




where ∂*I*_D_ is the differential source‐drain current, ∂*V*_G_ is the corresponding source‐gate voltage differential, *μ* is the electronic charge carrier mobility, C* is the volumetric capacitance, *W*, is the channel width, *d* is the channel thickness, *L* is the channel length, *V*_Th_ is the threshold voltage, and *V*_G_ is the gate voltage.^[16]^ All abovementioned parameters relevant to the OECT devices, including μC* product, and on/off current ratio are summarized in Table 4. Representative output curves obtained from inDTP‐T and saturation‐regime transfer curves of all OECT devices were plotted as shown in Figure 6a and b. All devices showed typical p‐type accumulation mode operation in which the drain current increased with the application of a negative gate potential (Figure S82). The two phenylene copolymers afforded slightly higher threshold voltages than the T and 2T copolymers; this trend was also supported by CV results (Figure 4, Table 3). For each polymer, OECTs were fabricated with channel lengths varying from 20 to 80 μm and the transconductance (*g*_m_) values obtained from each channel length is plotted as a function of (*Wd*/*L*)(*V*_Th_−*V*_G_) and shown in Figure 6c. All polymers show volume‐dependent transconductance increase, which is a typical characteristic of organic mixed ionic‐electronic conductors originating from ion permeability in the bulk film.^[33]^ From the slope of this plot, the μC* product, normalized by OECT channel dimensions and operating conditions, can be extracted to compare the mixed conduction properties of each polymer (Figure 6d).^[34]^ The average μC* product value recorded for inDTP‐T (267±52 F V^−1^ cm^−1^ s^−1^) was the highest value among the polymer series investigated here, which is on par with previously reported μC* value of p(g2T‐TT) (261 F V^−1^ cm^−1^ s^−1^).^[26]^

**Table 4:** OECT performance metrics.

Polymer	*d* [nm]	*g*_m_ [μS]	C*^[a]^ [F cm^−3^]	*μ*_OECT_^[b]^ [cm^2^ V^−1^ s^−1^]	*V*_Th_ [V]	μC*^[c]^ [F V^−1^ cm^−1^ s^−1^]	On/off
inDTP‐P	96	254±96	147±13	0.26	−0.58	38±10	10^4^
outDTP‐P	82	314±82	113±12	0.61	−0.63	69±21	10^4^
inDTP‐T	37	963±37	162±16	1.65	−0.55	267±52	10^4^
outDTP‐T	18	105±18	117±10	0.38	−0.49	45±15	10^2^
inDTP‐2T	70	853±70	168±14	0.68	−0.56	115±19	10^4^
outDTP‐2T	54	505±54	166±15	0.39	−0.50	65±19	10^4^

[a] Values calculated from the Bode plot obtained from EIS measurements; [b] Calculated from the transistor saturation mobility using the respective C* values; [c] Average value obtained from six channels from the slope of *g*_m_ as a function of (*Wd*/*L*)(*V*_Th_−*V*_G_).

To correlate the OECT performance with the structural properties of the corresponding polymers, it is necessary to decouple the *μ* value from μC*, which is an important material property related to the structure of the material.^[35]^ Accordingly, the C* values of the polymers were determined by electrochemical impedance spectroscopy (EIS). Detailed measurement setup and results are shown in the Supporting Information (Supporting Information Section 11 and Figure S83–S84). The peak C* values measured for each polymer are displayed in Figure 6e and Table 4. The C* values of the polymer films increase as the comonomer changes from P to T and 2T regardless of the side chain orientation (in vs out). This can be attributed to the increasing content of electron‐rich thiophene units in the polymer backbone.^[31]^

The hole mobilities of the polymers were calculated and displayed in Figure 6f and Table 4. These *μ* values were obtained by dividing μC* with C*, therefore representing the *μ* under the structural characteristics of a swollen state of polymer. inDTP‐T afforded the highest hole mobility of 1.65 cm^2^ V^−1^ s^−1^, while the other polymers yielded lower values ranging from 0.26 to 0.68 cm^2^ V^−1^ s^−1^. The trend of *μ* value is very similar to the trend observed from the μC* (Figure 6d). This clearly shows that, in this polymer series, the material's key factor determining OECT performance is the charge carrier mobility, not the capacitance. Despite the molecular weight variations of the polymers discussed above, it is worth emphasising that we do not find any indications that the μ and μC* trends are obscured by molecular weight effects. For instance, while inDTP‐T and inDTP‐2T both outperform their “out” regioisomer, one displays a higher molecular weight and the other a lower molecular weight than their corresponding “out” regioisomer.

### Computational simulations

**Figure 7:**
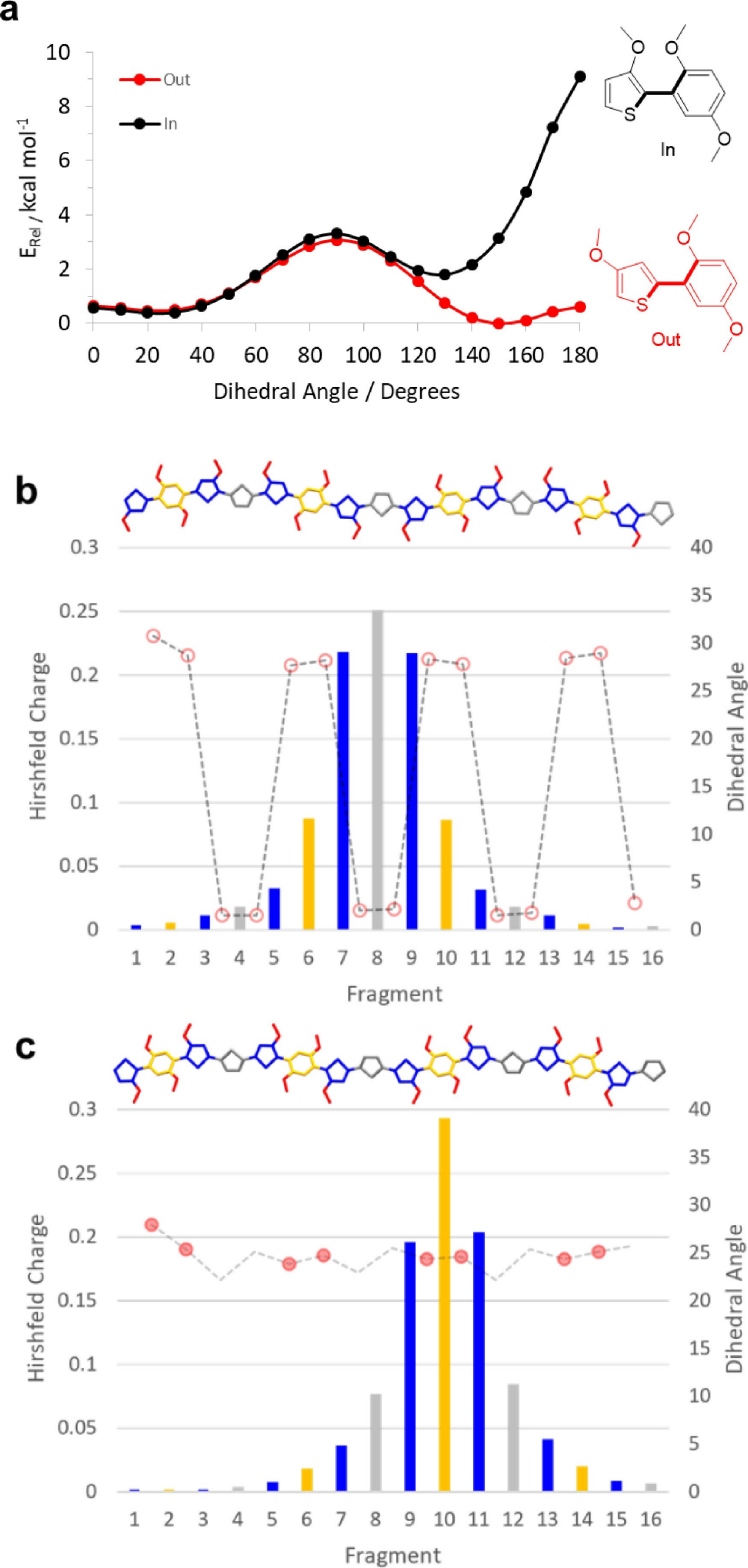
Computational simulation data. a) Torsional potential energy surfaces for model “in” and “out” segments. b), c) Hirshfeld charge distribution (left axis and bar chart) by fragment (blue=glycolated thiophene; orange=glycolated phenylene; grey=comonomer unit) and dihedral angles along optimised polymer chains (right axis, line plot) for outDTP‐T (b) and inDTP‐T (c) polymers with two intramolecular O−X interactions supporting the dihedral configuration denoted by filled circles, one O−X interaction denoted by open circles and no O−X interactions denoted by no circle.

Density functional theory (DFT) calculations were performed to investigate further the structural and electronic properties of the polymer series. In regard to the comparison of the “in” and “out” regioisomers and the impact on polymer backbone conformations, potential energy surfaces were compared for the two “in” and “out” motifs depicted in Figure 7a. While the “out” regioisomer shows two energy minima around dihedrals of 25° (S−O interaction) and 150° (O−H interaction), the “in” regioisomer shows a strong preference for the confirmation with approximately 25° that avoids a strongly repulsive O−O interaction. This is likely to lead to a higher degree of conformational disorder for the outDTP polymers compared to the corresponding inDTP derivatives, which in turn could result in a higher degree of energetic disorder and explain the lower hole mobility values observed for outDTP‐T and out‐DTP‐2T relative to the inDTP counterparts (Figure 6f).

Using the most energetically favourable conformations, oligomers comprising four repeat units were subsequently constructed to represent polymer structures, and the geometries optimized (*ω*B97XD/6‐31G*). The three inDTP polymers all have fairly constant dihedral angles along the polymer backbone in the range 20–30° with inDTP‐P having slightly larger dihedrals around the unsubstituted phenylene unit as expected (Figure 7c and Figure S85). For the “out” series, a similar conformational profile is observed for outDTP‐P, whereas outDTP‐T and outDTP‐2T show significantly larger variations with dihedral angles along the backbone varying from 0–30° (Figure 7b and Figure S85). This could potentially affect the structural organisation of the polymers in the solid state as will be discussed in the following section.

Electronic properties were investigated through the use of a tuned range separated functional approach (Table 5). From this, optimal omega values can be used to indicate the degree of electronic delocalisation along the polymer backbone with smaller values representing a higher degree of delocalisation. No clear trend is observed when comparing optimal omega values with *ω* being similar for the two regioisomers in the case of DTP‐P, *ω* being smaller for the “out” regioisomer of the T copolymer and larger for the 2T copolymer when comparing to the corresponding “in” version. Finally, we divided the studied oligomers into fragments and studied the positive polaron distributions through Hirshfeld charge calculations (Figure 7b–c, Table 5 and Figure S85). For all six polymers, a large fraction of the positive charge is always located on the two flanking glycolated thiophene units, irrespective of regioisomer and comonomer unit. For the remaining polaronic charge, a noteworthy difference is observed when comparing the “in” and “out” regioisomers with significantly more charge residing on the glycolated phenylene unit for the “in” polymers and more charge residing on the unsubstituted comonomer unit for the “out” series. This difference is most pronounced for the T and 2T copolymers and could again be a contributing factor behind the observed hole mobility differences observed assuming that the highly electron‐rich phenylene motif is better at stabilising the positive charge.

**Table 5:** Computed electronic properties.

Polymer	Optimal *ω* [Bohr^−1^]^[a]^	IP [eV]^[b]^	P^+^ [gT%]^[b,c]^	P^+^ [gP%]^[b,c]^
inDTP‐P	0.1165	5.05	52	31
outDTP‐P	0.1153	5.09	55	20
inDTP‐T	0.1172	4.87	50	33
outDTP‐T	0.1063	4.71	53	18
inDTP‐2T	0.1096	4.86	44	31
outDTP‐2T	0.1134	4.85	40	13

[a] *ω*B97XD/6‐31G* in gas phase; [b] Optimized *ω*B97XD/6‐31G* in chloroform polarizable continuum; [c] Percent of charge stabilized on the glycolated thiophene (gT) and glycolated phenylene (gP) fragments in positively charged systems, analysed by Hirshfeld atomic charges.

### Structural properties

Polymer thin films spin‐cast from chloroform solution were analysed using grazing incidence wide‐angle X‐ray scattering (GIWAXS) to study the structural organization of the polymer series as summarized in Table 6 with the corresponding 2D diffractograms and in‐plane and out‐of‐plane line cuts available in the Supporting Information (Figure S86). The out‐of‐plane peaks at low q‐values for outDTP‐P, inDTP‐T, and inDTP‐2T are assigned as (100) lamellar stacking peaks with further appearance of higher order peaks;[^36^, ^37^] meanwhile, out‐of‐plane peaks at q‐values around 1.5 Å^−1^ for outDTP‐T and outDTP‐2T are ascribed to π‐π stacking.[^38^, ^39^, ^40^] this indicates that the dominant molecular orientation of outDTP‐P, inDTP‐T, and inDTP‐2T is edge‐on, while outDTP‐T and outDTP‐2T are oriented face‐on relative to the substrate. In the case of inDTP‐P, no strong orientational preference is found.

**Table 6:** Solid state packing parameters extracted from the GIWAXS measurements.

	Lamellar Stacking (100)			π‐π stacking (010)		Unit chain length (001)		
Polymer	*q* [Å^−1^]	*d* [Å]	*L_c_* [Å]	*q* [Å^−1^]	*d* [Å]	*q* [Å^−1^]	*d* [Å]	Texture
InDTP‐P	0.344	18.3	74.8	1.469	4.28			Mixed
OutDTP‐P	0.396	15.9	82.0	1.449	4.34	0.410	15.3	Edge‐on
InDTP‐T	0.393	16.0	98.9	1.475	4.26	0.407	15.4	Edge‐on
OutDTP‐T	0.282	22.3	41.8	1.661	3.78	0.410	15.3	Face‐on
InDTP‐2T	0.382	16.5	94.1	1.490	4.22	0.418	15.0	Edge‐on
OutDTP‐2T	0.326	19.3	44.6	1.711	3.67			Face‐on

As discussed above, the coplanarity of the polymer backbone increases slightly when going from phenylene to the T or 2T comonomer unit due to the smaller ring size, lower aromaticity and fewer space‐filling hydrogens of thiophene compared to phenylene.[^41^, ^42^, ^43^] This is reflected in shorter π‐stacking distances for DTP‐T and DTP‐2T compared to DTP‐P for both regioisomers. The effect is particularly pronounced for the “out” series with outDTP‐T and outDTP‐2T both showing significantly shorter π‐stacking distances than any of the other four polymers (Table 6). We ascribe this to the additional intramolecular S−O close contacts enhancing the backbone coplanarity and interchain interactions (Figure 1) leading to shorter π‐stacking distances in the solid state.[^44^, ^45^, ^46^] The tighter π‐stacking, in turn, leads to more linearly extended glycolated side chains and larger lamellar spacings as also seen in Table 6.[^47^, ^48^, ^49^, ^50^]

Interestingly, the regiochemistry and choice of comonomer unit affect the dominant molecular orientation, leading in the case of outDTP‐T and outDTP‐2T to a face‐on dominant orientation as reported previously for other semiconducting polymers displaying a high degree of backbone planarity.[^44^, ^46^] It is noteworthy that outDTP‐T and outDTP‐2T, while having some completely coplanar fragments due to the engineered S−O interactions, are also expected to display a large degree of conformational disorder due to the two minima in the potential energy surfaces. With pre‐aggregation in solution often quoted as the driving force for the edge‐on orientation, we hypothesise that this conformational disorder is likely to prevent effective pre‐aggregation and therefore drive the face‐on orientation during film deposition in agreement with recent work on other semiconducting polymers.^[31]^

To further understand the dry‐state structural properties, the (100) peak coherence length (*L*_c_) is calculated for each polymer (Table 6 and Figure S87). Most significantly, outDTP‐T and outDTP‐2T, highlighted above for their enhanced conformational disorder and lower OECT charge carrier mobility, show markedly shorter coherence lengths than the corresponding “in” regioisomers.[^51^, ^52^]

**Figure 8:**
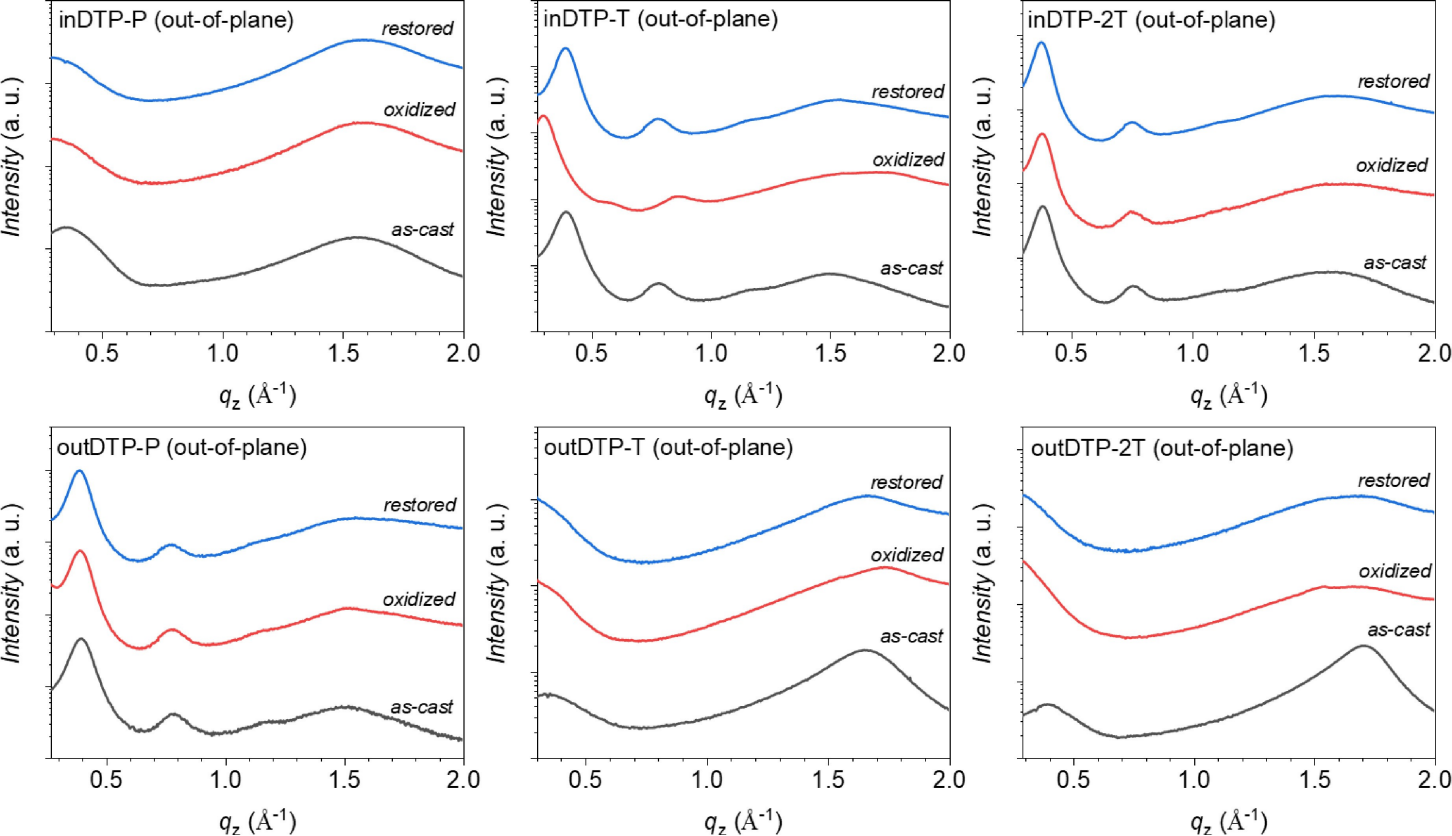
Out‐of‐plane (q_*z*_) line‐cuts of the 2D GIWAXS data obtained from polymer thin films spin‐cast from chloroform onto ITO‐coated glass; GIWAXS data was collected on the as‐cast films (black lines), after electrochemical oxidation (red lines), and after restoring the oxidized films to neutral (blue lines) as further described in the Supporting Information. The X‐ray wavelength was 1.11794 Å (*E*=11.09 keV), and the incidence angle of the beam light was ≈0.1°. The sample‐to‐detector distance was adjusted to be 220 mm.

While dry‐state GIWAXS characterisation does provide some structural insight that has been correlated with observed mixed ionic‐electronic properties probed by OECT devices,[^24^, ^53^] the swollen state created in the channel of an operating OECT device does not necessarily reflect the dry state accurately.[^25^, ^54^] Therefore, following initial GIWAXS measurements discussed above, we oxidized the polymer thin films electrochemically in 0.1 M aqueous NaCl under conditions mimicking the OECT operation and repeated the GIWAXS measurements, after which the films were finally reduced back to neutral electrochemically and subjected to GIWAXS again. The resulting data is displayed in Figure 8 (out‐of‐plane line cuts) and Figure S88–S91 (2D patterns and line cuts).

The three polymers displaying a predominant edge‐on orientation, outDTP‐P, inDTP‐T, and inDTP‐2T, all preserve their structural organisation after the electrochemical cycle (oxidation followed by reduction back to neutral) with clear lamellar peaks (100 and 200) observed before and after at unchanged q‐values. The highest performing OECT material, inDTP‐T, furthermore displays clear peaks from a new microstructure in the oxidized state, evidenced by out‐of‐plane lamellar peaks (Figure 8) and an in‐plane π‐stacking peak (Figure S91) corroborating elongated lamellae (≈21 Å) and tighter π‐stacks (≈3.5 Å) compared to the microstructure of the neutral film. The two other edge‐on polymers have a more unperturbed microstructure irrespective of the oxidation state. The three polymers with either face‐on or mixed orientation, outDTP‐T, outDTP‐2T, and inDTP‐P, experience a clear deterioration with respect to their semicrystalline microstructure when subjected to the electrochemical cycling; the distinct scattering peaks disappear upon oxidation and are not recovered when the film is reduced back to neutral.

This ex situ GIWAXS study indicates that the best‐performing OECT material, inDTP‐T, undergoes a reversible structural phase transition, possibly by accommodating polaronic charge‐compensating ions from the electrolyte in the glycolated side chain region.^[25]^ No such behaviour is seen for any of the other polymers, although the preservation of an ordered microstructure after CV cycling seems to correlate with slightly better overall OECT performance as seen for outDTP‐P and inDTP‐2T.

To further understand the polymers’ interaction with the aqueous electrolyte, we performed electrochemical quartz crystal microbalance with dissipation of energy (eQCM‐D) measurements on polymer films cast directly on the eQCM‐D sensors. Immersing the polymer films in 0.1 M aqueous NaCl, we observed passive swelling in the range 11–16 % (Figure S92, Table S4) in agreement with previous observations on related systems.^[21]^ Upon applying a bias of 0.8 V to oxidise the polymer films, the mass uptake of water and ions was in good agreement with the volumetric capacitance of each polymer (Figure S93, Table S4). For instance, inDTP‐T increased its mass by 3075 ng cm^−2^ upon oxidation whereas outDTP‐T only increased by 2530 ng cm^−2^ corroborating the differences in C* values (162 F cm^−3^ and 117 F cm^−3^, respectively). Considering the dissipation of energy recorded for the polymers, we observed trends that could be correlated to the OECT charge carrier mobilities. Despite the large mass uptake of inDTP‐T and inDTP‐2T upon oxidation, these two polymers showed small dissipation shifts, indicative of smaller changes in the rigidity of the polymer films during doping (Figure S93).^[21]^ This is in good agreement with inDTP‐T showing the highest hole mobility followed by inDTP‐2T.

## Conclusion

A series of six new polymers based on a new building block comprising a triethylene glycol substituted benzene flanked by triethylene glycol substituted thiophenes was synthesised by copolymerisation with phenylene, thiophene and bithiophene. Importantly, we altered the side chain orientation of the flanking thiophenes to study the influence of regiochemistry on the structural and mixed ionic and electronic conducting properties of this system in the context of OECT applications. We found that the OECT figure‐of‐merit can be improved by a factor of 7 through careful choice of regiochemistry and comonomer unit.

Solution UV/Vis spectroscopy indicated more disordered polymer conformations and lower effective conjugation lengths for the “in” regioisomers compared to their “out” counterparts, although this effect was largely diminished in the solid state with optical band gaps mainly governed by choice of comonomer unit rather than choice of regioisomer. Electrochemical studies showed low ionisation potentials with good switching in aqueous electrolyte as required for p‐type mixed conductors; somewhat lower ionisation potentials were observed for the “out” regioisomers ascribed to more coplanar backbone conformations due to a higher number of facile intramolecular S−O interactions compared to the corresponding “in” derivatives. Spectroelectrochemical measurements in aqueous electrolyte subsequently confirmed the ability to oxidise the bulk polymer thin film effectively and reversibly for all materials; this was evidenced by quenching of the π‐π* optical transition with the simultaneous formation of polaron absorption features.

GIWAXS measurements supported by DFT calculations were used to investigate the structural properties in the solid state. On the one hand, the “out” regioisomers were found to adopt more coplanar conformations as expected due to the S−O interactions introduced by molecular design. This in turn led to markedly shorter π‐stacking distances which should be favourable for efficient charge transport. On the other hand, conformational disorder was evident for the “out” regioisomers with both the “syn” and “anti” coplanar conformations being energetically favourable. We expect this disorder to prevent strong interchain interactions and pre‐aggregation during film formation. This was experimentally manifested by face‐on molecular orientations for outDTP‐T and outDTP‐2T and significantly shorter crystalline coherence lengths for these two polymers compared to the rest of the series. Conversely, the inDTP regioisomer displayed a “locked” conformation with one coplanar conformation much preferred energetically over the other. Ex situ GIWAXS, exploring the pristine, oxidized, and reduced polymer films, revealed significant differences across the polymer series. inDTP‐T was distinct by displaying a reversible structural phase transition upon oxidation, while e.g., outDTP‐T and outDTP‐2T showed significant loss of semicrystalline character upon electrochemical cycling.

OECT characterisation showed that all the prepared polymers are efficient p‐type mixed ionic and electronic conductors with μC* values ranging from 38 to 267 F V^−1^ cm^−1^ s^−1^. Upon decoupling of the volumetric capacitance and the hole mobility, we found that the variation in μC* across the polymer series is mainly governed by variations in hole mobility. We furthermore observed a strong correlation between crystalline coherence length and hole mobility thus placing inDTP‐T as the best‐performing material (267 F V^−1^ cm^−1^ s^−1^) followed by inDTP‐2T with a μC* value of 115 F V^−1^ cm^−1^ s^−1^. In contrast, no correlation was observed between π‐stacking distances and charge transport properties. Rather than maximising planarising intramolecular interactions and thereby enhancing close intermolecular π‐stacking distances (“out” regioisomers), it is thus clear that conformational “locking” (“in” regioisomers) and the associated longer‐range crystalline order have a stronger positive influence on the mixed ionic and electronic conduction properties. It should also be noted that while the higher performing inDTP polymers had a relatively large fraction of the polaronic charge residing on the glycolated phenylene unit, the outDTP derivatives had more polaronic charge stabilised on the comonomer unit; this could also play a role in explaining the lower hole mobilities extracted from the outDTP‐based OECTs.

In conclusion, control of regiochemistry is emerging as a powerful molecular design tool to control polymer backbone planarity and polaronic charge distribution. This was also shown recently in a related study on the well‐established g2T‐TT mixed conductor.^[21]^ Although thiophene regiochemistry has long been a pivotal focal point in the design of hydrophobic thiophene semiconductors for instance for field‐effect transistor and photovoltaic applications, the molecular design considerations are somewhat different for these very electron‐rich and hydrophilic thiophene‐based materials where both the head‐to‐head and head‐to‐tail conformation in principle can lead to coplanar conformation due to the direct oxygen linkage on the thiophene ring. Here, we have shown that focusing solely on maximising backbone planarity and extending the polaronic charge distribution across the largest fraction of the polymer backbone does not necessarily lead to the best performance. It is crucial during the molecular design phase to balance these considerations against the possibilities of introducing undesirable conformational disorder which will impede long‐range registry and therefore efficient charge transport over device‐relevant length scales.

## Conflict of interest

The authors declare no conflict of interest.

1

## Supporting information

As a service to our authors and readers, this journal provides supporting information supplied by the authors. Such materials are peer reviewed and may be re‐organized for online delivery, but are not copy‐edited or typeset. Technical support issues arising from supporting information (other than missing files) should be addressed to the authors.

Supporting Information

## Data Availability

The data that support the findings of this study are available from the corresponding author upon reasonable request.
